# A Rare Coexistence of Antineutrophil Cytoplasmic Antibody (ANCA)-Associated Vasculitis and IgG4-Related Disease: A Case Report and Literature Review

**DOI:** 10.7759/cureus.89738

**Published:** 2025-08-10

**Authors:** Zainulabdeen S Al-saedi, Lina Alatta, Mohammed A Miqdad, Hasan Hulwi, Oscar Rodriguez, Mourhege Alsaloum, Sheng Kuo

**Affiliations:** 1 Medicine, New York Presbyterian Queens Hospital, Flushing, USA; 2 Nephrology, New York Presbyterian Queens Hospital, Flushing, USA; 3 Research, Michigan State University, East Lansing, USA

**Keywords:** acute kidney injury, anca-associated vasculitis, igg4-related disease, interstitial nephritis, plasma-cell-rich infiltrates

## Abstract

Autoimmune kidney diseases can present with overlapping clinical and pathological features, posing diagnostic and therapeutic challenges. Elderly patients with comorbidities are particularly vulnerable to atypical or coexisting disease presentations. We present a rare case of an 83-year-old male patient with acute kidney injury (AKI) and multiple comorbidities, diagnosed with both antineutrophil cytoplasmic antibody (ANCA)-associated vasculitis (AAV) and immunoglobulin G4-related disease (IgG4-RD). The patient exhibited characteristic features of AAV, including positive P-ANCA and elevated myeloperoxidase (MPO)-ANCA titers, confirmed by biopsy showing multifocal necrotizing vasculitis and chronic-active interstitial nephritis. Additionally, the biopsy revealed plasma-cell-rich interstitial nephritis with numerous IgG4-positive plasma cells, suggesting the coexistence of IgG4-RD. The diagnosis was complicated by the patient’s baseline chronic kidney disease (CKD) and immunosuppressive therapy. This case highlights the diagnostic challenges in distinguishing AAV from IgG4-RD, particularly in elderly patients with comorbidities. While IgG4-RD is increasingly recognized as a cause of tubulointerstitial nephritis (TIN), its coexistence with AAV remains rare. This case underscores the importance of a high index of suspicion for diagnosing and managing complex, immune-mediated diseases in elderly patients.

## Introduction

Antineutrophil cytoplasmic antibody (ANCA)-associated vasculitis (AAV) is an autoimmune disorder characterized by the presence of ANCAs, which affect multiple organs, primarily the kidneys and respiratory system [1]. The diagnosis of AAV typically includes positive ANCA serology [2]. The hallmark features include rapidly progressive glomerulonephritis, pulmonary hemorrhage, and systemic inflammation [3]. Immunoglobulin G4-related disease (IgG4-RD) is a slowly progressing, indolent systemic, fibroinflammatory condition characterized by elevated serum IgG4 levels, tissue infiltration by IgG4-positive plasma cells, and fibrosis [4]. While the disease can affect almost any organ system, it commonly involves the pancreas, bile ducts, salivary glands, and kidneys [5]. Renal involvement in IgG4-RD can manifest as tubulointerstitial nephritis (TIN) [6]. The coexistence of AAV and IgG4-RD is rare but has been reported in several case studies, highlighting the diagnostic challenges posed by these conditions' overlapping clinical features [7].

## Case presentation

An 83-year-old male patient with a past medical history of stage IIIb chronic kidney disease (CKD) with a baseline creatinine of 1.7 mg/dL, type II diabetes mellitus, hyperlipidemia, hypertension, benign prostatic hyperplasia, liver cirrhosis attributed to hemochromatosis, prior hepatitis B infection, and pulmonary fibrosis with a usual interstitial pneumonia/idiopathic pulmonary fibrosis pattern on imaging presented to the emergency department with a few-week history of fatigue, weight loss, and poor appetite, without associated fever, rash, hemoptysis, hematuria, chest pain, or dyspnea. There was no history of relevant offending medications, and he was not on hydralazine for hypertension. On examination, vital signs were stable, with no respiratory distress or skin rash. Urinary obstruction was ruled out by ultrasound, which showed mildly echogenic kidneys of normal size.

Relevant laboratory testing is shown in Tables 1, 2. A kidney biopsy was performed due to worsening renal function and elevated myeloperoxidase (MPO) antibodies concerning for AAV. Of note, anemia in the 9-10 g/dL range was present at baseline, and prior urinalysis showed no hematuria but trace to 30 mg/dL proteinuria.

**Table 1:** Initial relevant laboratory tests WBC: white blood cells; MCV: mean cell volume; MCH: mean cell hemoglobin; ESR: erythrocyte sedimentation rate; Na: sodium, K: potassium; CO2: bicarbonate; BUN: blood urea nitrogen; Cr: creatinine; Ca: calcium; RBC: red blood cells; PCR: protein creatinine ratio

Complete blood count		
Labs	Value	Reference range
WBC	11.36	4.8-10.8 x 10 (3)/uL
Hemoglobin	9.3	13.3-17.7 g/dL
MCV	91.5	80-100 fL
MCH	28.1	26-34 pg
Platelet	329	150-400 x 10 (3)/uL
Neutrophils	8.86	1.8-8.5 x 10 (3)/uL
Lymphocytes	0.72	0.8-3.5 x 10 (3)/uL
Eosinophils	0.98	0-0.6 x 10 (3)/uL
Basophils	0.04	0-0.3 x 10 (3)/uL
ESR	102	0-20 mm/hr
Metabolic panel		
Labs	Value	Reference range
Na	135	136-145 mmol/L
K	3.5	3.5-5.1 mmol/L
CO2	19	22-29 mmol/L
BUN	42.9	8-23 mg/dL
Cr	2.32	0.67-1.17 mg/dL
Glucose	101	74-107 mg/dL
Anion gap	14	5-17
Ca	9.2	8.6-10.4 mg/dL
Phosphorus	2.1	2.5-4.5 mg/dL
Urinalysis		
Labs	Value	Reference range
RBC	24	0-2/HPF
WBC	4	0-3/HPF
Protein	100	Negative/trace mg/dL
Blood	Moderate	Negative
Cast	Negative	Negative
PH	5.5	5-8
Glucose	500	Negative
Ketone	Negative	Negative
Bilirubin	Negative	Negative
PCR	1,719.4 mg/g	<11 mg/g

**Table 2:** Relevant serological and immunological tests ANA: antinuclear antibodies; Anti DSDNA: anti-double-stranded DNA; RF: rheumatoid factor; anti-CCP: anticyclic citrullinated peptide; MPO: myeloperoxidase; ANCAs: antineutrophilic cytoplasmic antibodies; PR3: proteinase 3; GBM: glomerular basement membrane; HbSAg: hepatitis B surface antigens; HbSAb: hepatitis B surface antibodies; HbCAb: hepatitis B core antibodies; HCV Ab: hepatitis C antibodies; FLC K/L: free light chain kappa/lambda; C: complement

Lab	Value	Reference range
ANA	1:320	<1:80
Anti-dsDNA	<1:10	<1:10
RF	<8.4	0-14 IU/mL
Anti-CCP	5	<20: negative
C-ANCA	Positive 1:20	<1:20
P-ANCA	Positive 1:1280	<1:20
PR3 Ab	0	0-19 AU/mL
MPO Ab	125	0-19 AU/mL
Anti-RO-SSA	4	0-40 AU/mL
Anti-LA-SSB	0	0-40 AU/mL
Anti-centromere	3	0-40 AU/mL
Anti-SCL-70	1	0-40 AU/mL
Anti-GBM	0	0-19 AU/mL
HbSAg	Nonreactive	Nonreactive
HbSAb	64.37	<3.5
HbCAb	Reactive	Nonreactive
HCV Ab	Nonreactive	Nonreactive
Cryoglobulins	Negative	Negative
FLC K/L	0.99	0.26-1.65
C3	92	82-185 mg/dL
C4	27	15-53 mg/dL
CH50	61.1	38.7-89.9 U/mL

Pathology report

Light Microscopy

**Figure 1:**
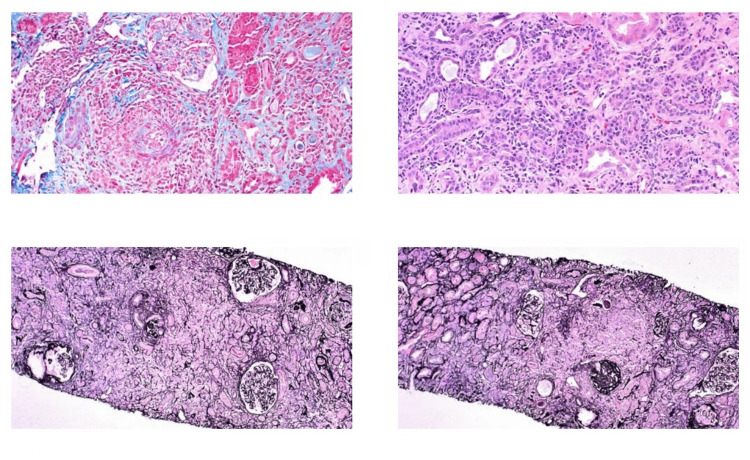
Histopathological findings by light microscopy

**Figure 2:**
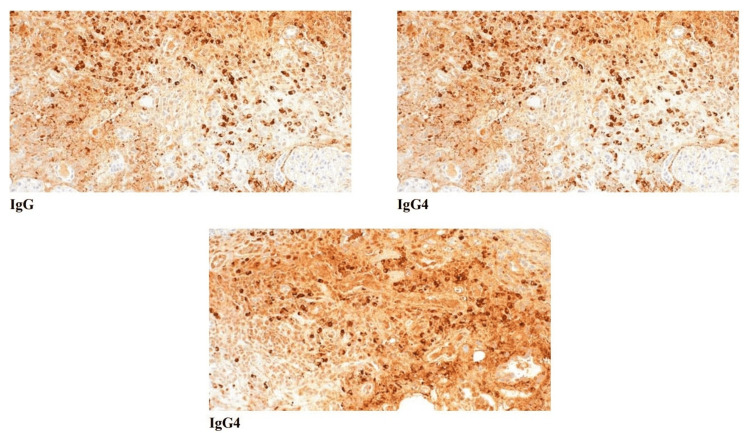
Histopathological findings by Light Microscopy showing IgG4 staining

The kidney biopsy sample shows 45 glomeruli, five of which are globally sclerosed and one segmentally sclerosed. Three glomeruli exhibit ischemic collapse. There are no crescentic lesions or endocapillary hypercellularity. The mesangium is mildly expanded by extracellular matrix. Tubules show degenerative changes, and the interstitium is inflamed with a dense, plasma cell-rich infiltrate, mild edema, and moderate tubulitis. Severe sclerosis is observed in arteries and arterioles, with focal hyaline degeneration and necrotizing vasculitis in the interlobular arteries (Figures 1, 2).

Immunofluorescence

**Figure 3:**
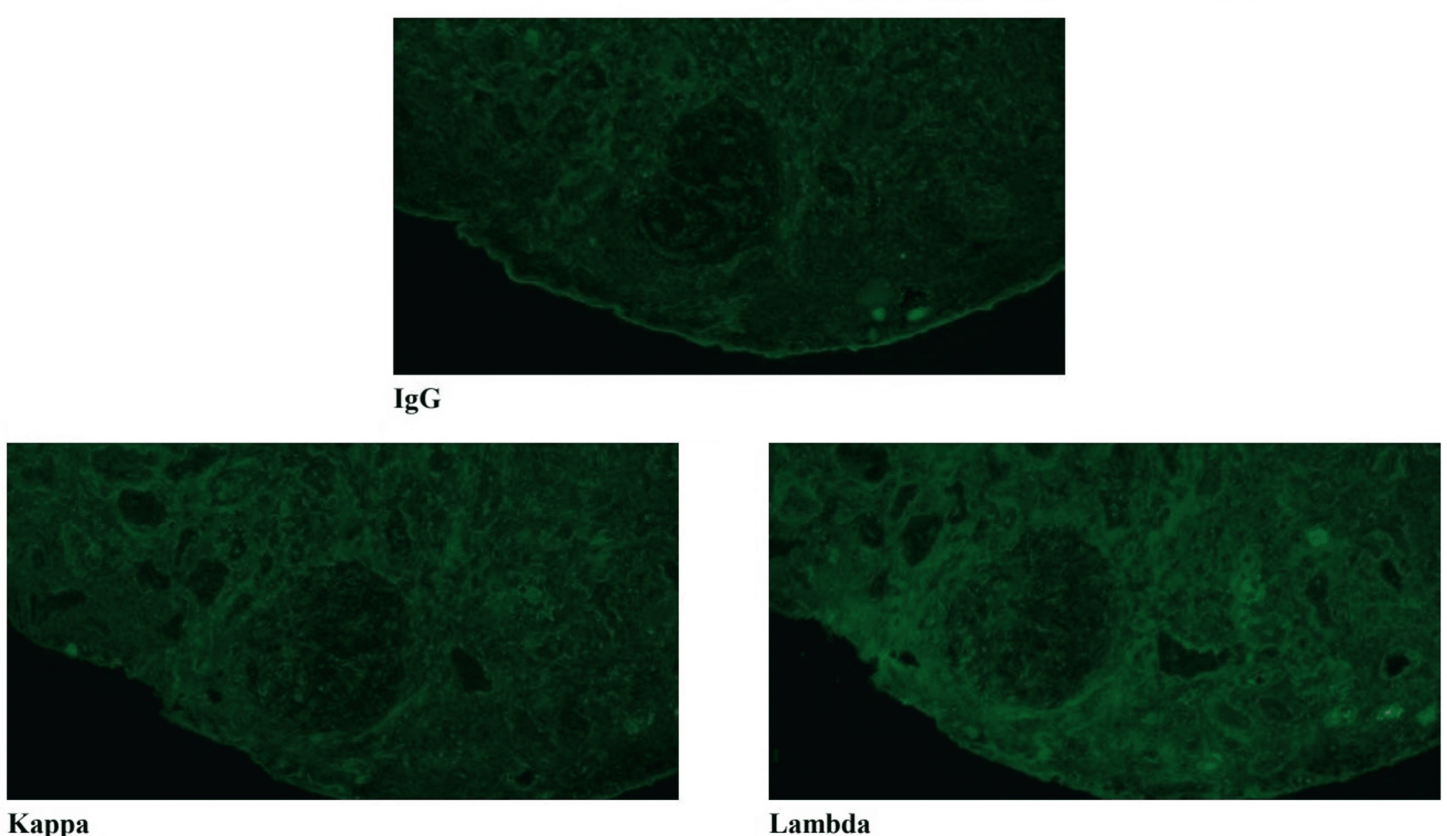
Immunofluorescence microscopy

The glomeruli show no significant reactivity for immunoglobulins or complement components, consistent with a pauci-immune pattern. Focal linear reactivity for C3 is observed in the tubular basement membranes. Intraluminal casts within the tubules are reactive for polyclonal IgA. The interstitium shows scattered fibrin deposits, along with mildly increased background staining for lambda light chains. Immunohistochemistry reveals both T and B lymphocytes within the infiltrate, along with numerous IgG- and IgG4-positive plasma cells (over 70/hpf) (Figure 3).

Electron Microscopy

**Figure 4:**
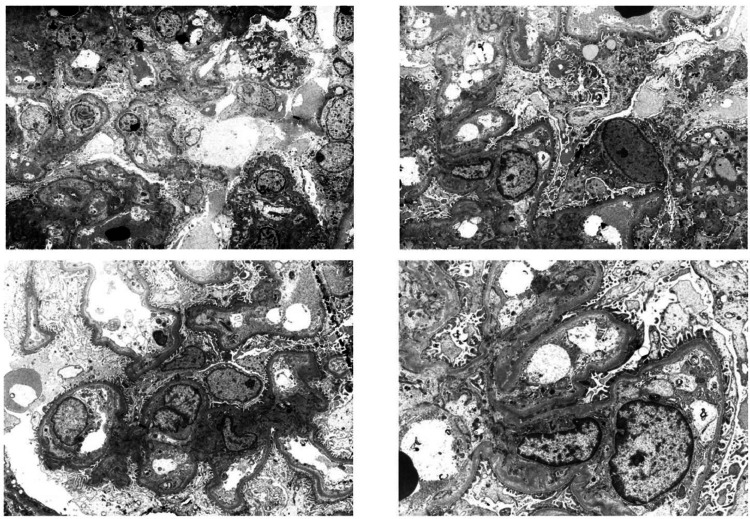
Electron microscopy

The examination reveals minimal segmental effacement of foot processes in the glomeruli, with wrinkling of the glomerular basement membranes. No electron-dense deposits are seen along the basement membranes or within the mesangium. The mesangium shows increased cellularity and mild matrix expansion. No tubuloreticular inclusions are noted in the glomerular endothelial cells, and a few capillary lumens are occluded by inflammatory cells (Figure 4).

Given the pathology findings, the serum IgG4 level was obtained and found to be 118.1 mg/dL (reference range: 4.0-86.0 mg/dL). After the initial pulse steroid therapy, he received cyclophosphamide induction, followed by a transition to rituximab and a steroid taper, with appropriate prophylactic measures.

## Discussion

AAV is an autoimmune disorder characterized by the presence of ANCAs, which target neutrophils and lead to systemic inflammation affecting multiple organs, primarily the kidneys and respiratory system [1]. The clinical diagnosis of AAV typically includes positive ANCA testing, with the two main antibody targets being proteinase 3 (PR3) and MPO [2]. Clinical manifestations of AAV can vary widely, but hallmark features include rapidly progressive glomerulonephritis, pulmonary hemorrhage, and systemic inflammation [3].

The pathogenesis of AAV begins with neutrophil-driven necrotizing inflammation, which causes vascular damage and leads to neutrophil apoptosis and necrosis. This injury results in bleeding and the release of plasma proteins, including coagulation factors, which are activated by cellular and tissue debris [1]. Tissue factor also promotes fibrin formation in areas of necrosis. As the process progresses, segmental necrosis is followed by crescentic formation. Over time, the initial neutrophil-rich inflammation transitions to one dominated by monocytes, macrophages, and T cells, with eosinophils often present. Eventually, fibrosis and scarring develop [1]. Isolated interstitial nephritis is a rare manifestation of AAV. Previous reports suggest that peritubular capillaritis contributes to interstitial fibrosis in AAV, potentially influencing long-term renal outcomes. Additionally, although granuloma formation is uncommon in renal AAV, it has been reported and may suggest histologic overlap with granulomatous diseases such as granulomatosis with polyangiitis (GPA) [8].

IgG4-RD is a systemic fibroinflammatory condition characterized by elevated serum IgG4 levels, tissue infiltration by IgG4-positive plasma cells, and fibrosis [4]. While the disease can affect almost any organ system, it commonly involves the pancreas, bile ducts, salivary glands, and kidneys [5]. Renal involvement in IgG4-RD often manifests as TIN, which can be challenging to distinguish from other forms of interstitial nephritis or autoimmune diseases such as AAV [5]. The diagnosis of IgG4-RD requires both histopathological evidence of tissue infiltration by IgG4-positive plasma cells and elevated serum IgG4 levels, although the latter is not diagnostic on its own [6].

IgG4-RD induces renal inflammation primarily through a T-helper 2 (Th2) and regulatory T-cell (Treg)-mediated immune response, leading to TIN rather than glomerular disease [9]. Infiltration by IgG4-secreting plasma cells, eosinophils, and M2 macrophages results in the release of cytokines such as interleukin-4 (IL-4), IL-10, and transforming growth factor-beta (TGF-β), promoting fibrosis, tissue remodeling, and immune tolerance rather than direct cytotoxicity [10]. Unlike autoimmune diseases that activate the classical complement pathway, IgG4 antibodies are functionally monovalent and do not trigger complement-mediated glomerular injury, thereby limiting direct damage to capillary loops [11]. Instead, chronic peritubular inflammation, storiform fibrosis, and obliterative phlebitis develop due to dysregulated fibroblast activation and vascular remodeling [12]. Additionally, altered Treg activity suppresses pro-inflammatory pathways, allowing persistent fibrosis without overt immune complex deposition [13]. While membranous nephropathy may occur due to subepithelial IgG4 immune complex deposition, the predominant renal manifestation remains TIN and CKD [13]. This contrasts with AAV, in which neutrophil activation, NETosis, and endothelial injury drive glomerular capillaritis and necrosis, resulting in acute glomerulonephritis [11].

A unique feature of IgG4 is Fab-arm exchange, a process in which IgG4 antibodies swap one of their antigen-binding arms with another IgG4 antibody, forming bispecific antibodies that are less likely to cause immune activation. However, it remains unclear how frequently this occurs in IgG4-RD, and if a substantial portion of IgG4 antibodies retain their original binding specificity, they may still contribute to inflammation [4,14].

The coexistence of AAV and IgG4-RD is rare but has been documented in several case reports. These cases highlight the diagnostic challenges associated with overlapping clinical features such as multiorgan involvement and systemic inflammation. Laboratory inflammatory markers and serum IgG4 levels may be elevated in both conditions. However, they possess distinct pathophysiologies; AAV is characterized by immune complex-mediated vasculitis, whereas IgG4-RD involves chronic inflammation and fibrosis [7,14,15]. Chronic AAV results from persistent inflammation and immune-mediated damage, even after treatment, leading to fibrosis and scarring, and ultimately to CKD or end-stage renal disease (ESRD). It is driven by ANCA, which activates neutrophils and triggers NETosis, resulting in endothelial and glomerular damage [10]. In contrast, IgG4-RD is marked by fibrosis and tissue remodeling primarily mediated by Th2 cells and Tregs, without immune complex deposition [9]. While both conditions can cause kidney injury and elevated inflammatory markers, AAV is defined by neutrophil-mediated vascular injury, whereas IgG4-RD is characterized by chronic peritubular inflammation and fibrosis [10,16,17].

Management of both conditions requires immunosuppression. Evidence-based treatment guidelines are well-established for AAV but remain limited for IgG4-RD due to its rarity. Ongoing clinical research is aimed at developing more targeted therapies based on a deeper understanding of the pathophysiology to improve patient outcomes [17-20].

Accurate diagnosis is essential, as treatment regimens for AAV and IgG4-RD differ significantly. AAV typically requires more aggressive immunosuppressive therapy to induce remission [21,22]. Current first-line treatment for AAV includes glucocorticoids in combination with immunosuppressive agents such as cyclophosphamide or rituximab [23]. Rituximab has been shown to be as effective as cyclophosphamide in treating patients with severe renal disease or alveolar hemorrhage, without notable differences in adverse event rates [3]. In contrast, IgG4-RD is often managed with less intensive immunosuppression, primarily corticosteroids [20].

In the presented case, the diagnosis of AAV was supported by clinical findings, positive ANCA testing, and histology. Although the patient had a history of diabetes mellitus and was initially presumed to have diabetic nephropathy as the cause of his CKD, no diabetic changes were observed on biopsy. It is conceivable that IgG4-RD was the underlying cause of his CKD all along, with superimposed AAV as the trigger for AKI on CKD. Without a kidney biopsy, the diagnosis of IgG4-RD would likely have been missed. This case further emphasizes the importance of maintaining a high index of suspicion for IgG4-RD, given its indolent course [21].

The decision to treat with high-dose corticosteroids and rituximab for the AAV component was consistent with current guidelines, while management of the IgG4-RD component was also considered [23,24]. Rituximab has demonstrated efficacy in IgG4-RD. In one study of 10 patients, nine showed significant clinical improvement within one month of treatment. One patient with advanced thyroid fibrosis (Riedel’s thyroiditis) had no thyroid improvement but showed no disease progression. All patients were able to discontinue prednisone and disease-modifying antirheumatic drugs (DMARDs) after rituximab. Only IgG4 levels significantly decreased. Four patients required retreatment after six months due to symptom recurrence or rising IgG4 levels; subsequent courses were effective and further reduced IgG4 concentrations. Elevated IgG4 at presentation was a reliable marker of disease activity [24].

## Conclusions

This case underscores the rarity and complexity of diagnosing and managing the coexistence of ANCA-associated vasculitis and IgG4-RD, particularly in elderly patients with multiple comorbidities. The overlapping clinical and histopathological features of these two diseases highlight the importance of maintaining a high index of suspicion when evaluating cases with renal involvement as part of multisystem manifestations, as well as the necessity of a thorough diagnostic workup, including renal biopsy and serological testing. While immunosuppressive therapy remains the cornerstone of treatment for both conditions, careful management is required to minimize adverse effects and to guide treatment plans and long-term prognosis. Further research is needed to better understand the pathophysiological mechanisms underlying the coexistence of AAV and IgG4-RD and to develop more effective diagnostic and therapeutic strategies for these challenging cases.
